# Effect of Schneiderian membrane perforation on sinus lift graft outcome using two different donor sites: a retrospective study of 105 maxillary sinus elevation procedures

**DOI:** 10.3205/iprs000090

**Published:** 2016-03-02

**Authors:** Andreas Sakkas, Ioannis Konstantinidis, Karsten Winter, Alexander Schramm, Frank Wilde

**Affiliations:** 1 Department of Oral and Maxillofacial Surgery, Facial Plastic Surgery, Military Hospital Ulm and Academic Hospital University Ulm, Germany; 2 Department of Prosthodontics, University of Dresden, Germany; 3 Institute of Anatomy, Medical Faculty, University of Leipzig, Germany

**Keywords:** sinus lift, membrane perforation, autogenous bone, maxillary atrophy, maxillary sinus grafts

## Abstract

**Background:** Sinuslift is meanwhile an established method of bone augmentation in the posterior maxilla. Aim of the study was to evaluate the significance of intraoperative Schneiderian membrane perforations during maxillary sinus floor elevation surgery using autogenous bone harvested from two different donor sites using a Safescraper device on the success rate, graft survival and implant integration.

**Methods:** The investigators conducted a retrospective cohort study at the Department of Oral and Maxillofacial Surgery of Military Hospital Ulm composed of patients with severe maxillary atrophy who underwent sinus augmentation from January 2011 until December 2011. Ninety-nine consecutive patients (89 men, 10 women) with a mean age of 43.1 years underwent sinus graft procedures in a 2-stage procedure using the lateral wall approach, as described by Tatum (1986). Data on patient age, smoking status, donor site and surgical complications were recorded and the relationship between Schneiderian membrane perforation and complication rate was evaluated. Dental implants were inserted 4 months after grafting.

**Results:** A total of 105 sinus lift procedures were performed in 99 patients. Sixty-one patients (61.6%) underwent sinus elevation with autogenous bone from the buccal sinus wall, while 38 patients (38.4%) bone harvesting from the iliac crest. Intraoperative perforation of the Schneiderian membrane was observed in 11 of the 105 sinuses (10.4%). These perforations resulted in 4 (36.3%) of the cases in major postoperative complications accompanied by swelling and wound infection. Membrane perforations were slightly associated with the appearance of postoperative complications (p=0.0762). In 2.4% of all cases, regarding 2 patients the final rehabilitation with dental implants was not possible because of extensive bone resorption.

**Conclusion:** Intraoperative complications performing sinus augmentation may lead to postoperative complications. With careful clinical and radiographic evaluation and appropriate treatment, the complications and risk for graft material displacement and implant loss can be eliminated.

## Zusammenfassung

**Hintergrund:** Die Sinusbodenelevation ist mittlerweile eine etablierte Methode zur Knochenaugmentation im seitlichen Oberkiefer. Ziel der Arbeit war die Evaluation der Auswirkungen von intraoperativ aufgetretenen Perforationen der Schneider’schen Membran bei Sinusbodenelevationen mit autogenen Knochentransplantaten, gewonnen mit Safescraper von zwei verschiedenen Donorseiten, auf den klinischen Erfolg, die Transplantats-Überlebensrate und die Implantatosseointegration.

**Methoden:** In einer retrospektiven Kohortenstudie an der Abteilung für Mund-, Kiefer- und Gesichtschirurgie des Bundeswehrkrankenhauses Ulm wurden die Daten von Patienten mit ausgeprägter Oberkiefer-Atrophie untersucht, bei denen zwischen Januar 2011 und Dezember 2011 Sinusbodenelevationen durchgeführt wurden. Bei 99 Patienten (89 Männer, 10 Frauen) mit einem mittleren Alter von 43,1 Jahren wurden 105 Sinusbodenelevationen in einem zweizeitigen Vorgehen mit der Technik des lateralen Zugangs nach Tatum (1986) unternommen. Patientenparameter wie Alter, Rauchen, Spenderegion und chirurgische Komplikationen wurden erhoben und die Korrelation zwischen Sinusmembranenperforation und postoperativer Komplikationsrate wurde ausgewertet. Die Implantation erfolgte 4 Monate nach Sinusaugmentation.

**Ergebnisse:** Es wurden insgesamt 105 Sinusbodenelevationen bei 99 Patienten durchgeführt. Bei 61 Patienten (61,6%) wurde eine Augmentation mit autologen Knochenspänen von der lateralen Sinuswand und bei 38 Patienten (38,4%) von dem anterioren Beckenkammrand durchgeführt. Eine intraoperative Perforation der Schneider’schen Membran trat bei 11 der Fälle (10,4%) auf. Davon haben sich bei 4 (36,3%) der Fälle postoperativ schwerere Komplikationen wie Wundinfektionen und Abszesse entwickelt. Es wurde eine leichte signifikante Korrelation zwischen intraoperativer Membranperforation und Auftreten von postoperativen Komplikationen festgestellt (p=0,0762). Bei 2 Patienten (2,4%) war aufgrund der ausgeprägten Transplantatresorption keine Implantatversorgung im Verlauf möglich.

**Schlussfolgerung:** Intraoperativ aufgetretene Perforationen der Sinusmembran können zu postoperativen Komplikationen führen. Eine sorgfältige präoperative klinische und radiologische Diagnostik ist notwendig, um postoperative Komplikationen und Risiken für Transplantat-Dislokation und Implantatverlust zu minimieren.

## Introduction

Dental rehabilitation of partially or totally edentulous patients with dental implants has become common practice in modern dentistry with reliable long-term results [1]. Although in many cases there is no sufficient bone volume for the placement of dental implants in the posterior maxilla. Different treatment approaches such as placing of short, titled or zygomatic implants have been proposed in order to overcome the problem of the atrophic posterior maxilla [2]. However, placing of short implants does not always eliminate the need of sinus elevation, because a residual bone of at least 5 mm is required. Sinus elevations procedures with autogenous or synthetic bone material are chosen to enable the placement of standard length implants. Depending on the remaining bone at the sinus floor the surgeon has to choose between the one- and the two-stage approach for sinus elevation. The one-stage approach was described by Tatum [3] and refers to the transalveolar or lateral wall approach. The two-stage approach is indicated in cases of extreme atrophy of posterior maxilla. This technique was described by Boyne and James [4]. 

Despite some recent advances in bone-substitute technology, autogenous bone grafts still regards as the “gold standard” for augmentation procedures because of their osteoinductive, osteoconductive and nonimmunogenic potential, providing higher bone quality and implant stability [2], [5]. Donor sites for autogenous bone are generally the oral cavity, iliac crest, tibia and calvaria. When choosing the appropriate donor site, the size of the bone defect and the surgical risks associated with the harvesting procedure must be taken into account by the clinician [6], [7]. 

The most common complication of sinus augmentation is perforation of the Schneiderian membrane, with a reported rate from 10% to 60% [8], [9], [10], [11], [12], [13], [14], [15], [16], [17], [18], [19]. Perforations can occur when the lateral wall is being iatrogen infractured or due to irregularities of the sinus floor such as the presence of sinus septae [16]. It has also been suggested that previous sinus surgery, chronic sinus pathology and absence of alveolar bone are risk factors to perforations of the maxillary sinus [17]. 

Postoperative infection and sinusitis after sinus augmentation have been explained by obliteration of the ostium owing to hematoma, edem or graft dislocation [20], impaired sinus production and impaired ciliary function [15], [17], [21], [22]. Loss of the biological function of the membrane as barrier due to perforation can increase sinus bacteria invasion and infection [23], [24]. The importance of sinus membrane integrity to confine the particulate graft and prevent infection and for overall graft and implant success has been reported, but conflicting information exists in the literature [15], [16], [19]. Multiple studies have shown an association between membrane perforation and acute sinusitis or graft infection [4], [16], [25], [26], whereas others have shown no association between membrane integrity and infection [22], [27], [28], [29], [30]. Timmenga et al. (2003) showed that maxillary sinusitis as a complication of sinus floor augmentation is significantly higher in patients with a predisposition to sinusitis or a history of chronic sinusitis [13], [22]. Similarly, many have reported a correlation between membrane perforation and graft failure [13], [23], [31], [32], whereas other studies have shown no association [16], [27], [28], [29]. Intraoperative damage of the membrane is always threatens the coverage of the graft materials. Many methods have been advocated for treatment of perforation of the Schneiderian membrane during the sinus floor elevation and augmentation. However, there are no guidelines for the treatment of these complications.

The rationale of conducting this study is to increase evidence regarding the association between intraoperative iatrogenic perforations of the sinus membrane with eventual complication rates following the staged sinus elevation. The increased awareness of the clinician regarding this risk indicator will allow a more sophisticated patient selection increasing the predictability of this surgical intervention.

This study examines the complication rates after occurring on Schneiderian membrane perforations by sinus elevation procedures using autogenous bone from two donor sites. As intraoral donor site was used the lateral wall of the posterior maxilla area, whereas the iliac crest was the extraoral donor site. The aim of this retrospective analysis was to determine the association between the intraoperative perforated Schneiderian membrane and postoperative infectious complications, graft failure, and implant loss and to determine the significance of sinusitis and graft infection or graft failure. Patient age was also considered as risk factor.

## Methods

### Study design

All participants signed an informed consent and all study procedures were performed according to the principles of the Declaration of Helsinki for Medical studies. A retrospective analysis was performed following data collection. Reporting of the collected data is performed based on the recommendations of the Strengthening the Reporting of Observational studies in Epidemiology (STROBE).

The study population consisted of patients who underwent maxillary sinus augmentation from January 2011 until December 2011 at the Department of Oral and Maxillofacial Surgery of Ulm Military and Academic Hospital. Files of 99 patients were retrospectively reviewed and following data were collected: medical history of patient, periodontal status, smoking status, age of patient, date of maxillary sinus elevation and date and sort of postoperative complications, management of complications and data regarding implant placement. Surgical observations such as intraoperative membrane perforation, wound infection, abscess, hemorrhages and graft lost were documented as complications, regardless of eventual healing.

All surgical interventions were conducted by the same experienced surgeon, who is certificated according to the German Association of Oral Surgeons, who followed the standardized clinic protocol. The maxillary sinus elevation was conducted for patients with severe maxillary bone atrophy rated class V according to the Cawood and Howell classification [33], a residual maxillary sinus floor less than 5 mm high, and need of implant treatment.

In the initial patient consultation, medical and dental health history, age as well as smoking status were recorded. Additionally, comprehensive periodontal examination was conducted. Preoperative radiographic assessment included careful evaluation of any pathologic conditions of the sinus using orthopantomograms and maxillary computed tomography and measurement of the residual bone in the posterior maxilla. 

In ninety-three patients a unilateral and in six patients a bilateral sinus elevation procedure was performed. In regard to complications, the observation period was restricted to a healing time of 4–5 months after grafting until implant placement. 

### Surgical protocol 

The bone harvesting procedure was performed using a standardized surgical technique. The anesthesia of all patients was carried out with Ultracain^TM^ D-S (Hoechst Marion Roussel Deutschland, Frankfurt, Germany) containing 1:200,000 epinephrine into the buccal and palatal maxillary area. A single shot of 2.2 gr penicillin (Augmentan^®^, GlaxoSmithKline Consumer Healthcare GmbH & Co. KG) or, if penicillin allergic, 600 mg clindamycin (Clinda-saar^®^, MIP Pharma GmbH) as well as 250 mg prednisolon (Solu-Decortin^®^, Merck Pharma GmbH) was administered intravenous to patients 30 minutes prior to surgery. 

### Grafting from the lateral sinus wall

The incision was made on the top of the alveolar ridge, or slightly on the palatal side, through the keratinized attached mucosa. A mucoperiosteal flap was raised and the preparation started with the bone scraper (Safescraper; C.G.M. S.p.A., Divisione Medicale META, Italy). Bone from the lateral wall of the sinus was collected as part of the antrostomy. The preparation was finished with a large round diamond bur that cannot easily damage the membrane or perforate the bony wall. The Schneiderian membrane was carefully dissected and elevated using special mucosal sinus elevators. If small perforations appeared in the sinus membrane they were repaired with a collagen membrane. Bone from the maxillary buccal buttress was harvested with the bone scraper by pushing the end of the device toward the bone surface and simultaneously pulling the device backward. Collection of 2–3 ml of bone was feasible with a mean surgical time of 5 minutes for harvesting. The graft material was then placed in the sinus cavity and the bony sinus window was covered with a resorbable collagen membrane (Bio-Gide^®^, Geistlich Biomaterials, Baden-Baden, Germany). Finally, the mucoperiosteal flap was replaced and sutured. A radiographic control was always performed postoperatively to evaluate the outcome of the surgical procedure. 

### Grafting from the anterior iliac crest

In all cases, surgery was performed under general anesthesia. One hour before surgery, 2.2 gr penicillin and 250 mg prednisolon were administered intravenously. The iliac crest was exposed and autogenous grafts from the anterosuperior edge of the iliac wing were harvested with an oscillating saw, keeping a safe distance from the anterosuperior iliac spine. After osteotomy, the corticocancellous bone blocks were harvested using chisels. The sinus elevation was performed following the technique described above. 

### Postoperative management

Panoramic radiographs were always acquired for all patients postoperatively. Patients were instructed to rinse their mouth with chlorhexidine 0.2% for 2 to 3 weeks twice daily. After this period the sutures were removed. Patients were advised to avoid physical stress, blowing their noses, or sneezing for a period of three weeks. Removable, provisional prostheses were generously adjusted. No antibiotic therapy was continued after surgery and patients were instructed to use non-steroidal anti-inflammatory drugs only if pain was present. Routine follow-up was scheduled for 2 weeks when no complications occurred.

### Complications

Any patient with clinical signs of postoperative complication such as pain, inflammation of the operated area or nasal congestion combined with headache and fever was examined clinically. If any infection developed without fluctuance, amoxicillin/clavulanate (Augmentan^®^, GlaxoSmithKline Consumer Healthcare GmbH & Co. KG) (3 gr/day for 5 days) was prescribed. When fluctuance was present, incision and drainage was performed in local anesthesia. Persistent infection or signs of acute sinusitis symptoms required removal of the graft and flushing of the sinus in combination with antibiotics intravenously. Failure was defined as any sinus graft that secondarily required debridement and irrigation or failure of any implant within the grafted sinus before loading. A successful sinus graft had loaded implants with at least 1 year follow-up with no mobility or pain on function. 

### Dental implant treatment

After a healing period varying from 4 to 5 months after the grafting procedure, clinical ad radiographic evaluations were performed and implants were placed in a routine fashion. 

### Statistical analysis

Statistical analysis included descriptive statistics using SAS^®^ Software Version 9.3. A Fisher’s exact test was performed to determine the association between membrane integrity at the time of sinus lift augmentations and secondary infection and graft failure. A Chi-squared test was used to test the significance of the difference between morbidity and age. Descriptive statistics of the “complications” included relative and absolute frequencies. The significance level was set at p≤0.05.

## Results

### Patient population

Totally 105 sinus floor elevations were performed in 99 patients (89 men, 10 women) to treat severely atrophic maxilla using autogenous bone. The mean age was 43.1 ± 1.55 years (range, 28 to 61 years). Of the 99 patients receiving grafts, 23 were smokers (23.2%). Seven patients were pre-diagnosed with general-advanced periodontitis, which was successfully treated before bone grafting. Associated to pre-diagnosed sinus disease, five patients were recorded with chronic symptoms of sinusitis. 

In 61 patients, a sinus elevation was performed with autogenous bone from the buccal sinus wall, while 38 patients underwent bone harvesting from the iliac crest. Ninety-three of the patients were partially edentulous in the posterior maxilla, and only six were completely edentulous.

Of the 105 sinus floor elevations, 88 (83.8%) were defined absolutely successful and 17 (16.2%) had adverse effects, such as swelling, wound infection with pus exit, or acute sinusitis symptoms. The total complication rate was 26.6%. Intraoperative complications were observed in 10.4% (n=11) of the cases and postoperative complications in 16.1% (n=17). 

### Intraoperative membrane perforation

Of the 105 sinuses that met the stated inclusion criteria, 11 (10.4%) sinuses were perforated during augmentation. The perforations were then covered with a resorbable collagen membrane (Bio-Gide^®^, Geistlich Biomaterials, Baden-Baden, Germany) which applied as sealant to overlap the site of perforation prior to insertion of the graft material. Four out of 11 patients (36.3%), in whom the sinus membrane was perforated, experienced postoperative complications accompanied by swelling and wound infection. On the contrary, 13 of the 94 sinuses (13.8%) without intraoperative membrane perforation showed postoperatively abnormalities such as local wound dehiscence or abscess development. With regard to postoperative complications, the results show a slightly significant difference according to the iatrogen caused sinus membrane perforation (p=0.0762). 

### Postoperative infections/maxillary sinusitis 

In 17 patients suppuration was detected 1–3 weeks after surgical treatment (Table 1 (Tab. 1)). In eleven cases (10.4%) a local wound dehiscence without fluctuance developed 10–14 days after surgery. Overall, 27.2% (n=3) of the sinuses with local wound dehiscence had a membrane perforation during augmentation. These patients were treated with local surgical debridement and antibiotic therapy and scheduled to regular control appointments. The dehiscence healing was no later than 10 days uneventful by secondary granulation. Of the 4 sinuses (3.8%) developing an abscess postoperatively, one had an intraoperative membrane perforation during surgery. Once the infection was confirmed by clinical and radiographic examination, the sinuses were drainaged, and systemic antibiotics were administered. 

Symptoms of acute sinusitis such as nasal congestion, headache, diffuse pain on the operated facial site, fever or redness, were diagnosed in two patients one week after sinus floor elevation. No perforation of the sinus membrane occurred in these two patients intraoperatively. According to their medical history none of those patients had pathological findings of the sinus in the past. Despite the appropriate treatment with antibiotics, the bone grafts showed an extensive resorption. The wound healing was subsequently uneventful, but there was not enough bone for insertion of implants. 

### Sinus graft failure

There was an overall sinus graft failure rate of 1.9% (Table 2 (Tab. 2)). All the sinuses with perforated membranes at the time of graft placement were after eventful healing successfully treated with dental implants. 98.1% of sinus grafts were successful, as measured by implant loading of longer than 1 year. Of the two sinuses that had graft failure during the healing period, none of them had a perforated Schneiderian membrane. These two patients refused any additional surgical treatment. 

With regard to patient’s age, 5 cases of sinus membrane perforation were noticed in the group <40 years (10.0%) and 6 cases in the group ≥40 years (12.2%) (Table 3 (Tab. 3)). No significant difference between age and postoperative complications was found (p=0.7657). 

### Dental implant placement

Before positioning implants, after 4–5 months of healing, radiographic examination (orthopantomography or CT) showed good integration of the bone graft in all patients. A total of 179 dental implants were placed with satisfactory primary stability in the augmented areas. None of the implants were lost during the healing period and were osseointegrated at the time of implant exposure.

## Discussion

The objectives of this study were to evaluate the graft survival after 2-staged maxillary sinus elevation prior to implant placement, when intraoperative membrane perforation occurred. The main objective focused to assess the complication rates of this specific surgical intervention in patients who treated in a university clinic in Germany. Moreover, aim of this study was not to compare the implant survival but rather to outline the outcome of the augmentation procedure of the maxillary sinus by presenting the results of a large sample.

The literature states that the most common complication of sinus lift augmentation is the perforation of the Schneiderian membrane [8], [9], [10], [11], [12], [13], [14], [15], [16], [17] , [18], [19]. The rate of membrane perforation in this review was 10.4%. The present results showed a slight significant association between postoperative complications after sinus augmentation and membrane integrity (p=0.0762). Jodia et al. 2014 showed that in 15.3% of patients a perforation of the Schneiderian membrane occurred [34]. The membrane perforation could represent a window for bacterial penetration and invasion into the grafted area. The lower percentage in the present study can be related to the careful examination performed to ensure membrane integrity. Although the membrane perforation could represent a window for bacterial penetration and invasion into the grafted area, the authors did not find any correlation between the treatment outcome and the subsequent graft failure rate. 

In the literature, the following predisposing factors that can influence the chance of Schneiderian membrane perforation have been identified: anatomical variations, thickness of the membrane, previous sinus infection, former surgical treatments and surgeon’s experience [15], [35], [36], [37]. Anatomical predispositions consist of thickness of the lateral maxillary sinus wall, convex lateral sinus wall, formation of maxillary sinus septa and connection between Schneiderian membrane and oral mucosa [15], [35], [36], [37]. Despite accurate preoperative radiographic investigations, in some cases the laceration is unavoidable even when the surgical manoeuvres are performed at best [15], [35], [36]. Studies have suggested that overfilling of the maxillary sinus may cause necrosis of the membrane and secondary perforation with loss of graft into the sinus [22], [26], [27]. Disrupted mucociliary apparatus function and loss of the biologic barrier owing to perforation of the membrane can increase sinus bacteria invasion and infection [23], [24], explaining the increased secondary infection found in this study. The association between sinus perforation and graft dislodgement into the sinus and disruption of the normal sinus physiology has also been described in the literature [2], [15], [17]. 

The authors measured the success and failure of the graft and the implant survival until 1 year after loading. Failure was defined as a secondary removal of the augmented sinus graft, following no implant treatment. The overall failure rate of sinus lift augmentations in the present retrospective study was 1.9%. The survival rate of 98.1% was very high estimated in comparison to other study results. A systematic review by Wallace and Froum reported a 91.5% implant survival rate in the grafted sinus [38]. Ardekian et al. (2006) reported a success rate of 94% for implants placed in the augmented sinus at 4 years, with no significant difference between intact and perforated membranes [39]. Barone et al. (2006) reported an implant survival rate of 94.3% in augmented sinuses and no increased complication rate with sinus membrane perforations [11]. According to Moreno et al. 2014, the intraoperative damage of the Schneiderian membrane (25.7%) was not correlated with postoperative complications [35]. In most studies, no statistical difference was observed in the success rates of implants placed in augmented sinus regions in patients whose Schneiderian membrane was perforated versus those patients in whom the membrane remained intact. 

The present study did not show a greater occurrence of graft and implant failure when membranes were perforated at the time of augmentation. These results do not fit those of Shlomi et al. (2004), Nolan et al. (2014), Proussaefs et al. (2004), Khoury (1999), Hernández-Alfaro et al. (2008) [18], [19], [23], [31], [32]. Failures occurred in our study in 2.1% of the augmented sinuses with intact membranes compared with 0% of sinuses with intraoperative perforated membranes. 

There are many options for treating perforation of the Schneiderian membrane. Suggested surgical techniques to overcome these perforations include suturing, using fibrin adhesive, and overlapping with a resorbable collagen membrane [23]. Proussaefs et al. (2004) have suggested that a sinus membrane perforation larger than 2 mm could be associated with reduced implant success compared to sites where the sinus membrane was not perforated [23]. Within the limits of this study population, the 11 sinuses with membrane perforation showed slightly significant complications during the healing period, but without influence on the final failure rate. In maxillary sinus floor elevation procedures, it is essential to have good knowledge of the different anatomical and surgical findings, to minimize perioperative and postoperative complications. 

At our institution, we routinely use resorbable collagen membrane to cover the perforation irrespective to the size of the damage. Some studies report abandoning sinus lifting procedure when wide perforations cannot be repaired [30], [31]. Despite Hernández-Alfaro demonstration of an inverse relationship between the size of laceration and the implant survival, we suggest not to interrupt the surgical procedure [32].

The sinus graft is a relatively complex operation compared to the simple implant placement. The longer duration and the additional tissues and sinus space involved increase its potential for postoperative complications. Although, the sinus graft is considered to be a safe treatment modality in which complications are uncommon [27]. This statement was also due to our study confirmed, in which the final rehabilitation with dental implants was successful by 98.1% of the incidents. 

Interesting is the rather low complication rate of 1.9% for postoperative maxillary sinusitis in our study. Other authors have reported transient sinusitis in 10–26% of their patients [22], [26], [28]. Generally, patients who suffer from chronic sinusitis show significantly higher failure rates and have more postoperative complications [11]. Zijderveld et al. recorded a prevalence of 1% of postoperative maxillary sinusitis after sinus lift procedures, while Moreno et al. mentioned a frequency of 2.9% in their study [24], [35]. In the 2 cases of this study with postoperative maxillary sinusitis, the grafts had to be removed and the patients were not treated furthermore with implants. When the maxillary sinus is filled with blood, a delay of maxillary sinus clearance is thought to occur, because it is generally assumed that a reduction of the patency of the osteomeatal unit creates a potential risk for the development of maxillary sinusitis. It was reported that patients in whom postoperative chronic maxillary sinusitis occurred apparently have a predisposition for this condition [24]. However, our two patients did not show any sinus pathology in the preoperative diagnosis. Preoperative sinus disease has been positively correlated with the development of acute postoperative sinusitis after maxillary sinus harvesting [24]. However, more research should be conducted to determine how to perform a harvesting procedure in those patients without the risk of graft failure. Patients with a history of sinusitis should therefore be evaluated preoperatively to rule out factors related to sinus clearance which could be exacerbated by the normal inflammatory process produced by the sinus augmentation. Chronic sinusitis requires the administration of pharmacologic treatment, as prescribed by an otorhinolaryngologist, to provide the best possible surgical environment. 

No significant difference between age and postoperative complications existed (p=0.7657). Until now, no study has reported a higher rate of secondary complications after sinus elevation procedures in older patients. In the study of Nolan et al. (2014), the higher relative failure rate in older patients with intact membranes at augmentation might be explained by increased secondary membrane tear owing to poorer membrane vasculature and secondary necrosis, poorer healing potential, or poorer patient compliance in this group [19].

Based on a retrospective evaluation of 105 sinuses with 1 year of follow-up, the present data support the theory that sinus membrane perforation has a positive effect on the development of postoperative complications such as wound infections and acute sinusitis, but not on the implant failure rate. Although the authors did not investigate the size of the perforation and subsequent failure, some studies have found that success correlates inversely with the size of the perforation [32]. The ability to deal with perioperative perforation and postoperative complications remains imperative to overall graft success.

## Conclusions

This study demonstrates that intraoperative perforation of the Schneiderian membrane does not cause negative long-term effects on sinus bone grafts and dental implants. However, more studies correlating the size of sinus membrane perforation with the type of repair performed, as well as the contribution of patient’s age to the sinus morbidity after perforation are needed.

## Notes

### Competing interests

The authors declare that they have no competing interests.

## Acknowledgements

We thank Karsten Winter for his assistance in statistical data analyses and measurements and Professor Dr. Dr. Alexander Schramm for his permission and assistance with study administration. The author’s responsibilities were as follows: Andreas Sakkas, Ioannis Konstantinidis, Alexander Schramm, Frank Wilde: designed the research and wrote the manuscript; Karsten Winter: analysed statistical data; Andreas Sakkas, Ioannis Konstantinidis: edited the manuscript; Alexander Schramm, Frank Wilde: conducted the research and had primary responsibility for the final content of the manuscript; and all authors: read and approved the final manuscript. None of the authors had a conflict of interest.

## Figures and Tables

**Table 1:**
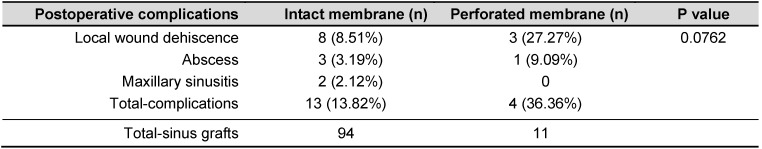
Membrane integrity at augmentation procedures with postoperative complications

**Table 2:**
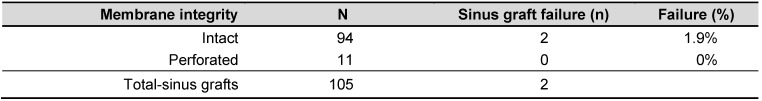
Membrane integrity at augmentation procedures of failed grafts

**Table 3:**
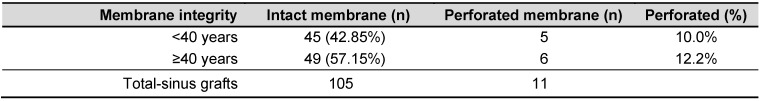
Membrane integrity at augmentation procedures with postoperative complications according to patient’s age
